# Tissue-Specific RNA Expression Marks Distant-Acting Developmental Enhancers

**DOI:** 10.1371/journal.pgen.1004610

**Published:** 2014-09-04

**Authors:** Han Wu, Alex S. Nord, Jennifer A. Akiyama, Malak Shoukry, Veena Afzal, Edward M. Rubin, Len A. Pennacchio, Axel Visel

**Affiliations:** 1 Lawrence Berkeley National Laboratory, Berkeley, California, United States of America; 2 U.S. Department of Energy Joint Genome Institute, Walnut Creek, California, United States of America; 3 School of Natural Sciences, University of California, Merced, California, United States of America; Stanford University School of Medicine, United States of America

## Abstract

Short non-coding transcripts can be transcribed from distant-acting transcriptional enhancer loci, but the prevalence of such enhancer RNAs (eRNAs) within the transcriptome, and the association of eRNA expression with tissue-specific enhancer activity *in vivo* remain poorly understood. Here, we investigated the expression dynamics of tissue-specific non-coding RNAs in embryonic mouse tissues *via* deep RNA sequencing. Overall, approximately 80% of validated *in vivo* enhancers show tissue-specific RNA expression that correlates with tissue-specific enhancer activity. Globally, we identified thousands of tissue-specifically transcribed non-coding regions (TSTRs) displaying various genomic hallmarks of bona fide enhancers. In transgenic mouse reporter assays, over half of tested TSTRs functioned as enhancers with reproducible activity in the predicted tissue. Together, our results demonstrate that tissue-specific eRNA expression is a common feature of *in vivo* enhancers, as well as a major source of extragenic transcription, and that eRNA expression signatures can be used to predict tissue-specific enhancers independent of known epigenomic enhancer marks.

## Author Summary

Up to 80% of mammalian genomes are actively transcribed, producing large numbers of non-coding RNAs without known functions. One particularly exciting category of such non-coding transcripts are the recently discovered enhancer RNAs (eRNAs) transcribed from distant-acting enhancer elements. Studies in cell-based paradigms suggest a functional requirement for such eRNA in enhancer-mediated gene regulation. In this study, we explored the *in vivo* expression dynamics of tissue-specific non-coding RNAs in embryonic mouse tissues *via* in-depth transcriptome profiling. Our results suggest that enhancers may be a predominant function associated with differentially expressed non-coding loci across developing tissues, and that differential eRNA expression signatures from total RNA-Seq can be used to identify uncharacterized tissue-specific *in vivo* enhancers independent of known epigenomic marks. Our results highlight the widespread and potentially important role of eRNAs in orchestrating gene expression and the necessity for functional studies in interpreting genome-wide enhancer predictions.

## Introduction

Development and function of mammalian tissues rely on the dynamic control of tissue-specific gene expression, a process largely regulated by distant-acting transcriptional enhancers [1]–[3]. Disruption of enhancer sequences can lead to severe phenotypes in mouse models [4]–[9]. Furthermore, population-scale genetic studies indicate that a large proportion of sequence variants associated with human diseases affect non-coding functions in the genome, of which enhancers are a major category [10]. Despite their functional relevance, the genome-scale identification of enhancers that are active *in vivo* in developmental and disease processes remains challenging. In principle, genome-wide profiling of enhancer-associated epigenomic marks (e.g. H3K27ac and CBP/p300) enables the genome-scale identification of enhancers predicted to be active in a given cell type or tissue [2], [3], [11]–[15]. However, none of these marks is unique to enhancer regions or found at all enhancers and ChIP-based technology has well-documented limitations with sensitivity and specificity [3], [14]–[16].

Recently, expression of short non-coding transcripts has been described as a feature of many enhancers with a possible tight correlation between cell type-specific enhancer activity and eRNA expression levels [17]–[20]. Using cap analysis of gene expression (CAGE) in a collection of human tissues and cell type, Andersson et al. [21] identified over 40,000 candidate enhancers marked by bidirectional capped RNA expression suggesting that RNA transcription can provide a complementary approach for *de novo* enhancer discovery. Anecdotal evidence suggests a functional requirement for such eRNAs in enhancer-mediated gene regulation [22], [23]. Regardless of the molecular mechanisms underlying eRNA-mediated regulatory functions, the prevalence of eRNA transcription at the whole transcriptome level *in vivo* and whether eRNA expression signatures can potentially be used as an independent mark for *in vivo* enhancer discovery remain poorly explored.

In this study, we compare eRNA expression profiles determined via total RNA sequencing across developmental mouse tissues and demonstrate highly tissue-specific genome-wide expression signatures of eRNAs *in vivo*. We find that eRNA expression globally correlates with tissue-specific enhancer activity and that RNAs transcribed from *in vivo* enhancers constitute a major proportion of tissue-specifically expressed non-coding RNAs. Finally, we demonstrate through application of reporter assays in transgenic mice that differential expression of eRNAs can correctly predict tissue-specific *in vivo* enhancer activities independent of other chromatin-associated marks.

## Results

### Tissue-specific eRNA expression in developing tissues

**Figure 1:**
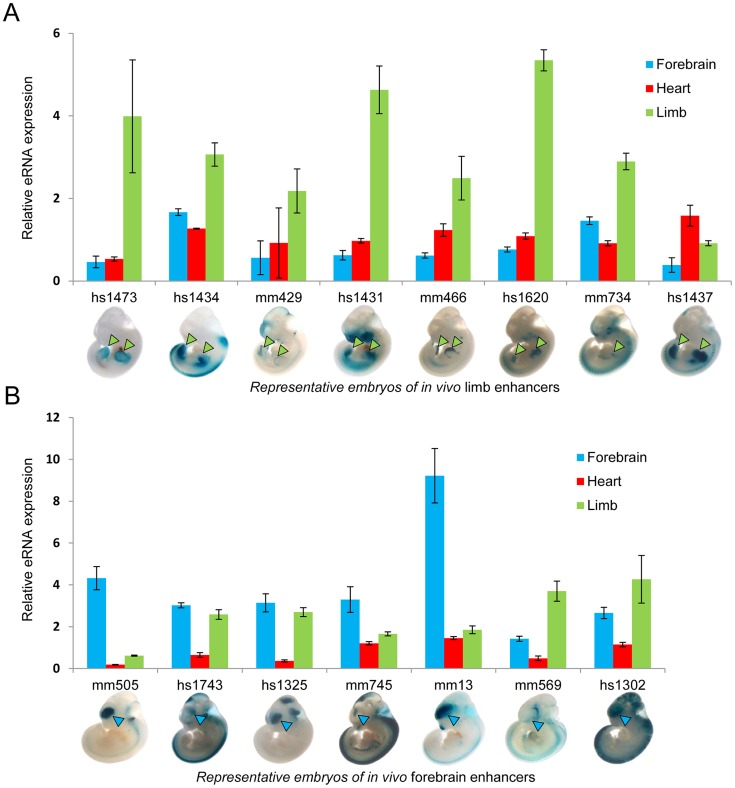
Tissue-specific eRNA expression at a subset of tissue-specific *in vivo* enhancers. (A) The expression of eRNAs was quantified by RT-PCR for 8 randomly selected known limb enhancers in three tissues. (B) Tissue-specific eRNA expression from 7 known forebrain-specific enhancers. The expression of eRNAs were quantified by RT-PCR for 7 randomly selected forebrain enhancers in three tissues. Results from triplicate experiments were plotted (forebrain: blue; heart: red; limb: green). Error bars represent SEM. Representative LacZ-stained embryos at E11.5 from transgenic assays for individual elements are shown at the bottom. Arrowheads indicate reproducible LacZ staining patters in limb (green) or forebrain (blue).

To test the hypothesis that eRNA transcription marks active *in vivo* enhancers in a tissue-specific manner, we first measured eRNA expression from 15 intergenic enhancers active in mouse embryonic forebrain or limb buds that were randomly selected from a larger collection of previously identified *in vivo* enhancers [24]. We assessed eRNA expression from each enhancer by quantitative RT-PCR across three different embryonic mouse tissues including forebrain, limb, and heart as a negative control (**Figure 1**). While baseline expression of each eRNA was detected in all three tissues, in 80% of cases eRNAs from tissue-specific enhancers showed highest expression in the predicted tissue compared with the other two tissues (12/15; p = 0.0006, Fisher's exact test), suggesting that eRNAs are commonly expressed from tissue-specific developmental enhancers with a quantitative relationship between eRNA transcription and tissue-specific enhancer activity.

**Figure 2:**
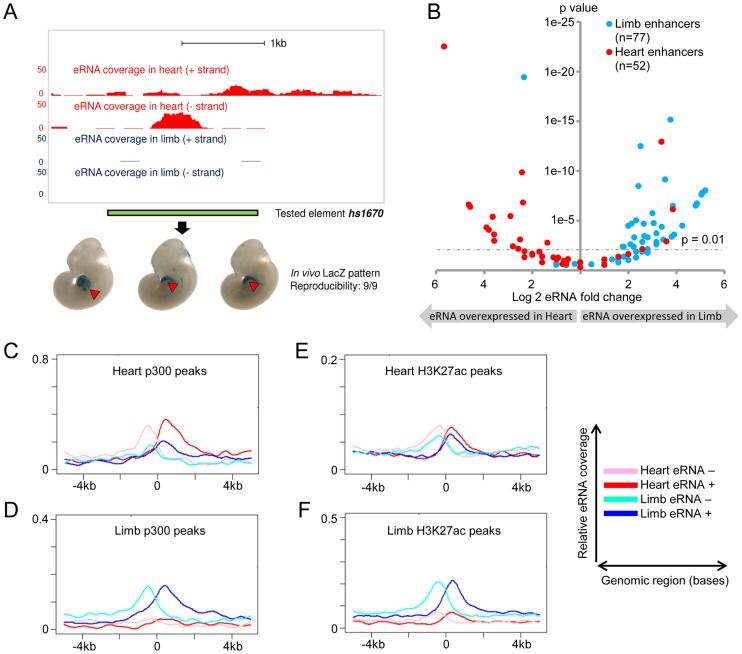
Global eRNA expression profiles. (A) Tissue- and strand-specific eRNA expression around a known heart enhancer (hs1670). Scales corresponding to read count are shown on the left. Genomic region cloned for the transgenic reporter assay is indicated by the green bar. Representative LacZ-stained embryos at E11.5 from transgenic assays for element hs1670 are shown at the bottom. Red arrowheads indicate reproducible LacZ staining pattern in heart. (B) Differential eRNA expression at known heart- or limb-specific enhancers correlates with the tissue-specificity of *in vivo* enhancer activities. Log2-transformed expression fold-changes of eRNAs arising from heart- (red) or limb-specific (cyan) enhancers are plotted against their associated p-value for each fold change (see **Methods**). (C–F) Cumulative strand-specific eRNA expression across candidate enhancers in a 10 kb window centered on p300 (C/D) or H3K27ac (E/F) ChIP-Seq peaks from the respective tissue. Sequencing reads mapped to forward strand (red in heart, blue in limb) or reverse strand (pink in heart, cyan in limb) are displayed separately.

To study eRNA expression from *in vivo* enhancers beyond this small-scale qPCR screen, we examined genome-wide total RNA transcription in embryonic heart and limb, two tissues with different developmental origins and trajectories, and with divergent *in vivo* enhancer landscapes as assessed by epigenomic marks [25]–[27]. We extracted total RNA from limb and heart tissues microdissected at mouse embryonic day [E] 11.5. Following ribosomal RNA depletion, we used a strand-specific total RNA sequencing protocol to generate more than 200 million sequencing reads from each tissue (see **Methods**, **Table S1**). While the majority of sequencing reads (53% in heart, 60% in limb) mapped to annotated mouse cDNA sequences, a considerable proportion (38% in heart, 30% in limb) mapped to introns as well as intergenic regions, consistent with a possible association with *in vivo* enhancers. Examination of individual genomic loci containing known enhancers revealed examples of bidirectional tissue-specific eRNA expression from validated intergenic and intragenic enhancers consistent with their *in vivo* activity (**Figure 2A** and **Figure S1**). These results indicate widespread transcription from non-coding sequences *in vivo* and anecdotally support correlation of *in vivo* enhancer activity with tissue-specific eRNA transcription.

### Expression of eRNA correlates with enhancer activity

In order to assess tissue-specific eRNA expression more systematically, we examined eRNA expression associated with a large collection of *in vivo*-validated tissue-specific enhancers [24], [26], [28] (http://enhancer.lbl.gov). To avoid confounding factors arising from the presence of pre-mRNAs, we restricted this analysis to intergenic *in vivo* enhancers (see **Methods**). We examined a total of 145 such enhancers that are active in heart or limb. In general, enhancers were substantially enriched in uniquely mapped reads, and they were nine times as likely as random non-coding regions to contain ten or more independent reads within 1 kb of the enhancer midpoint (p = 5.5E-108 based on background distribution; see **Table S2** and **Methods**). While 41% of enhancers met this stringent threshold, overall 92% of enhancers showed evidence of at least weak transcription (≥1 uniquely mapped reads; p = 2.3E-15 based on background distribution, see **Table S2** and **Methods**). Consistent with our small-scale sampling of enhancers by quantitative PCR (**Figure 1**), 79% of heart enhancers and 83% of limb enhancers showed higher eRNA expression in the tissue where enhancer activity was observed *in vivo* (**Figure 2B**; p<10^−8^, Fisher's exact test). We next examined tissue-derived RNA signatures at intergenic regions enriched for enhancer-associated p300 and H3K27ac epigenomic marks [27], [29] from the same tissues (see **Methods**). Similar to known *in vivo* enhancers, eRNA transcription was highly enriched around the center regions defined by ChIP-Seq, and tissue-specific eRNA expression patterns correlated with the predicted enhancer activity based on tissue-specific p300 or H3K27ac signature in the same tissues (**Figure 2C–F**; See **Methods**). This global correlation between tissue-specific eRNA expression and enhancer activity corroborates previous observations derived from CAGE analysis of human cell types and tissues [21] and supports the possibility that eRNA expression profiling from tissues may provide an effective approach for identifying tissue-specific *in vivo* enhancers.

### *De novo* discovery of tissue-specific non-coding RNA expression

**Figure 3:**
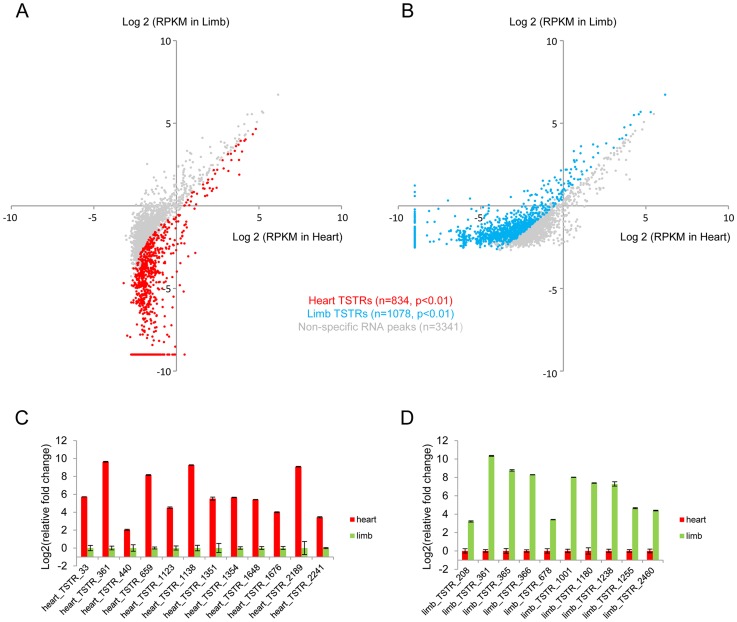
*De novo* identification of tissue-specifically transcribed regions. Dot plot showing all TSTRs identified by total RNA-Seq from heart (A) and limb (B) E11.5 tissues. Cyan and red dots indicate limb- or heart-specific TSTRs (p<0.01). Grey dots indicate RNA peaks without significant expression differences between the two tissues. RPKM<2^−9^ were arbitrarily set to 2^−9^ for visualization purposes (see **Methods**). A total of 22 candidate TSTRs were selected from heart (C) or limb (D) TSTRs. Tissue-specific RNA expression were quantified by RT-PCR by using total RNA samples from heart or limb tissues at E11.5 (see **Methods**). Error bars represent SEM.

To explore the potential of eRNA profiling for *de novo* enhancer discovery, we first used a sliding window approach to identify candidate intergenic regions enriched for RNA expression. Known coding and intronic regions and unannotated transcripts were removed, which led to the identification of 3,422 and 3,775 intergenic regions in heart and limb, respectively, that showed marked RNA expression at a conservatively chosen threshold of ≥10 uniquely mapped reads (see **Methods**; **Figure S2A–B** and **Table S2**). These regions included 834 heart-specific and 1,078 limb-specific loci (tissue-specifically transcribed regions, TSTRs) that were differentially expressed in these two tissues (**Figure 3A–B** and **Table S3**). Most of these ∼2,000 TSTRs were located distal to the nearest transcription start site (**Figure S2C**). There is substantial overlap between TSTRs identified from developing mouse tissues in this study and candidate transcription start sites (TSSs) captured by CAGE from mouse cells and tissues [30]. Overall, 45% of heart TSTRs and 55% of limb TSTRs overlap with at least one CAGE-derived TSS candidate. This represents a strong enrichment compared to random control sequences (8% and 8.3%, respectively; p<4.3E-68, Fisher's exact test, see **Methods**), but also indicates that large numbers of additional enhancer candidates were identified by analysis of *ex vivo* tissue at relevant developmental stages. Tissue-specific expression of a panel of 22 candidate TSTRs was tested and in all cases confirmed by quantitative RT-PCR (**Figure 3C–D**, see **Methods**), demonstrating that these RNA-seq data sets accurately identified non-coding TSTRs across tissues.

### TSTRs are associated with candidate *in vivo* enhancers

**Figure 4:**
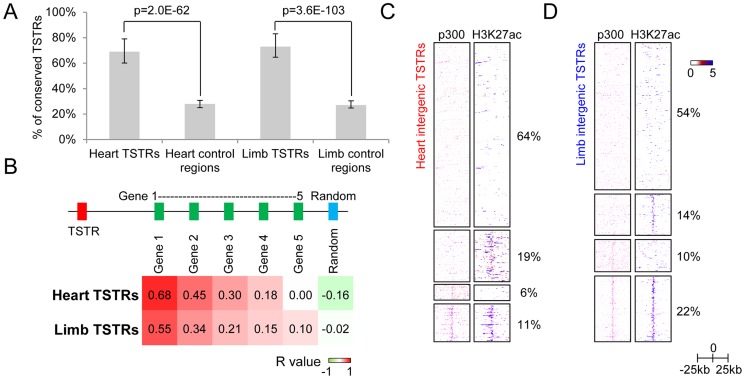
Intergenic regions marked by tissue-specific RNA expression may represent regulatory enhancer elements. (A) Fraction of TSTRs or random control regions (all size normalized to 1 kb from center) that are under strong evolutionary constraint (30 vertebrate phastCons; see **Methods**). Error bars represent 95% binomial proportion confidence interval. (B) Heatmap of Pearson correlation coefficient between tissue-specificity of TSTRs and nearby genes (see **Methods**). Genes 1 to 5 indicates the first to the fifth closest genes to the corresponding TSTR regardless of strand. For comparison, correlation with random genes on the same chromosome as the TSTR is shown. (C and D) Heatmap of p300 binding and H3K27ac signal within a −25 kb to +25 kb window surrounding the center of all heart TSTRs (C) or all limb TSTRs (D). Each line represents a single TSTR for individual tissues, and color scale indicates the normalized signal from individual ChIP-Seq experiment (see **Methods**).

To assess whether these TSTRs may represent *in vivo* enhancers, we first examined their evolutionary sequence constraint, a feature associated with many distant-acting enhancers [25], [31], [32]. We found that 69% and 73% of TSTRs in heart and limb, respectively, overlap with elements under evolutionary constraint as compared to 28% and 27% of random control sequences (p<2.0E-62, Fisher's exact test; **Figure 4A** and **Figure S2D**). Additionally, heart TSTRs are enriched near genes critical for cardiovascular and heart development, whereas limb TSTRs are enriched near genes involved in muscle tissue development and limb development/morphogenesis (**Table 1**). Heart and limb TSTRs are also enriched for different sets of transcription factor binding motifs related to development of the respective tissues compared with random genomic sequences (**Table S5** and **Table S6**). Finally, we compared tissue-specific TSTR expression with mRNA levels of nearby genes in two tissues (see **Methods**). The strongest correlation was observed between TSTRs and their nearest genes (Pearson correlation: R = 0.68 for heart, R = 0.55 for limb), and decreased substantially for more distant genes (**Figure 4B**). These results support that TSTRs may represent regulatory elements coordinating the transcription of nearby genes.

**Table 1:** Top 10 GO biological processes enriched in genes nearby TSTRs (sorted by p-value).

*Rank*	*GO Biological Processes*	*Binomial P-Value*	*Binomial Fold Enrichment*
***Heart-specific TSTRs***			
1	vasculature development	1.3E-30	3.0
2	blood vessel development	9.8E-29	3.0
3	cardiovascular system development	1.5E-28	2.5
4	blood vessel morphogenesis	3.0E-26	3.1
5	regulation of myotube differentiation	8.6E-23	25.9
6	cardiac muscle tissue development	2.6E-20	4.2
7	tube development	2.5E-19	2.4
8	cardiac myofibril assembly	3.1E-19	19.1
9	positive regulation of cardioblast differentiation	1.5E-18	22.1
10	tissue morphogenesis	2.8E-18	2.4
***Limb-specific TSTRs***			
1	regulation of skeletal muscle tissue development	1.7E-25	5.9
2	embryonic limb morphogenesis	1.7E-22	3.8
3	limb development	1.3E-20	3.1
4	regulation of striated muscle tissue development	1.8E-20	4.1
5	regulation of muscle organ development	3.5E-20	4.1
6	limb morphogenesis	1.3E-19	3.1
7	heart valve development	4.9E-19	6.5
8	negative regulation of response to DNA damage stimulus	7.5E-19	18.3
9	regulation of skeletal muscle fiber development	2.1E-18	5.7
10	regulation of cell development	1.0E-17	2.1

To evaluate the overlap of TSTRs with enhancer-associated epigenomic marks, we examined p300 and H3K27ac enrichment (**Figure 4C–D**). We find that 36% and 46% of heart and limb TSTRs are marked by p300 and/or H3K27ac. TSTRs with and without epigenomic enhancer marks show similar expression level and substantial evolutionary constraint (**Figure 4A** and **Figure S3A–B**). However, the transcription of TSTRs with enhancer marks tends to be more balanced in both directions, whereas TSTRs marked by tissue-specific RNAs only are more biased toward one direction (**Figure S3C–D**). In addition, TSTRs negative for p300 and/or H3K27ac are more distal to the nearest transcription start sites (**Figure S3E**). These results indicate a substantial overlap of extragenic TSTRs with enhancer-like regions. However, this does not exclude the possibility that subsets of the observed TSTRs represent other classes of regulatory elements or unannotated non-coding loci.

### Transgenic validation of enhancer predictions based on TSTRs

**Figure 5:**
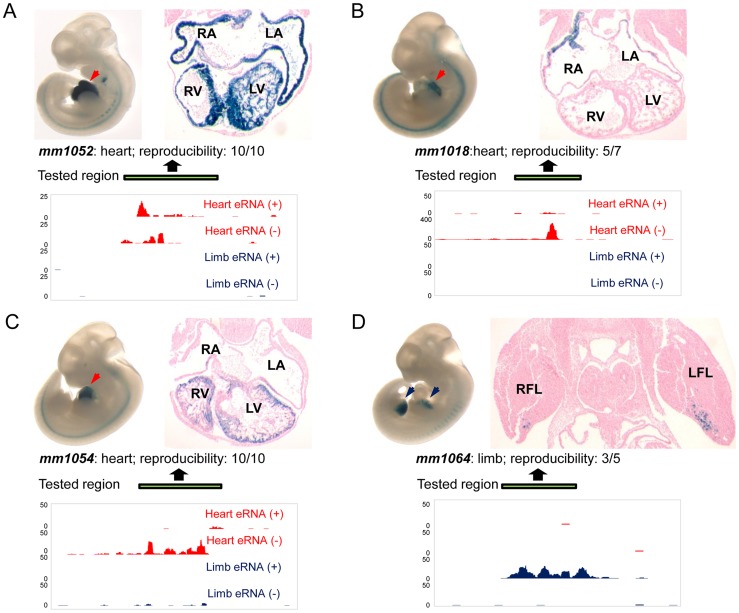
Transgenic characterization of TSTRs for tissue-specific enhancer activity. For each tested element, lateral views of whole-mount LacZ-stained embryos at E11.5 are shown in top left panels and transverse sections through heart or limb regions are shown in the top right panels. Arrowheads indicate reproducible LacZ staining pattern in heart (red) or limb (blue). Element ID and reproducibility of expression patterns are indicated at the bottom of the images. Strand-specific eRNA coverage of the tested regions in heart (red) or limb (blue) is shown in the bottom panels. Scales corresponding to read count are shown on the left of the coverage. Genomic regions cloned for the transgenic assay are indicated by green bars. (A) Enhancer element mm1052 with activity in both atrial and ventricular regions. (B) Enhancer element mm1018 shows activity in the right and left atrium. (C) Enhancer element 1054 with activity exclusively in the right and left ventricle. (D) Enhancer element mm1064 is active in the anterior domains of both forelimb and hindlimb, and only transverse section of forelimb is shown as an example. RA: right atrium; LA: left atrium; RV: right ventricle; LV: left ventricle; RFL; right forelimb; LFL: left forelimb. Transgenic results of all tested elements are available through the Vista Enhancer Browser (http://enhancer.lbl.gov).

To directly assess the potential of TSTRs identified by transcriptome profiling for the *de novo* discovery of tissue-specific *in vivo* enhancers, we used a transgenic mouse enhancer assay previously shown to reliably capture *in vivo* enhancer activity [25], [27], [33]. In an initial retrospective comparison, we found that heart- or limb-specific TSTRs overlap with 12 tested elements that had previously been examined due to increased conservation or enhancer associated epigenomic marks [24]. Of these elements, 9/12 (75%) were annotated as tissue-specific positive enhancers *in vivo* (**Table S7**, http://enhancer.lbl.gov). Next, we performed transgenic mouse assays for another set of 19 TSTRs that had not previously been tested (**Table S8**) and exhibited tissue-specific RNA expression. This panel included elements both with and without detectable p300 and/or H3K27ac signal in ChIP-Seq experiments (**Table S8**) that were chosen blind to the identity of nearby genes. Mouse genomic DNA for individual TSTRs with up to 2 kb of flanking sequence was cloned upstream of a minimal heat shock promoter fused to a *lacZ* reporter gene and transgenic mice were assayed by whole-mount staining for the expression of *lacZ* reporter at E11.5 [25] (see **Methods**). Only elements that drove reproducible reporter gene expression pattern in at least three embryos were considered positive enhancers. In total, 8/19 (42%) candidate enhancers predicted by tissue-specific RNA expression functioned as positive enhancers *in vivo* (**Figure 5**, **Table S8** and **Figure S4**). In all cases, the observed tissue-specific *in vivo* enhancer activity was consistent with the tissue specificity of the corresponding TSTR. As representative examples, transgenic whole-mount embryos and transverse sections for elements mm1052, mm1018, mm1054 and mm1064 are shown in **Figure 5**. In these examples, reproducible LacZ reporter activities were detected in both atrial and ventricular regions of the heart (**Figure 5A–C**) and anterior regions of the fore- and hindlimb (**Figure 5D**). Combining the results from newly performed enhancer assays and retrospective comparisons with pre-existing *in vivo* data sets, 17 of 31 TSTRs (55%) represented *in vivo* enhancers, and for 15 of these 17 enhancers (88%) the tissue specificity of eRNA expression correctly predicted the *in vivo* enhancer activity patterns. These results support the general utility of eRNA profiling as an informative mark for *in vivo* enhancer prediction.

## Discussion

Recent large-scale transcriptome studies suggest that up to 80% of mammalian genomes may be actively transcribed [34]–[37]. While many of these transcripts show differential expression signatures across cell types and tissues, the majority of non-coding transcripts have not been associated with *in vivo* functions. In the present study, we explored the *in vivo* expression dynamics of tissue-specific non-coding RNAs using a total RNA-Seq strategy that captures both coding and non-coding transcripts [18]. Our results suggest that the majority of enhancers show evidence of tissue-specific eRNA transcription. In addition, *de novo* identified tissue-specifically transcribed non-coding regions (TSTRs) showed major characteristics of canonical enhancers. These results indicate that enhancers are a predominant function associated with differentially expressed non-coding loci across developing tissues.

CAGE analysis from human cell lines and tissues showed that incorporating enhancer expression data can increase the validation rate of ENCODE enhancer predictions and that bidirectional capped RNA signatures can in principle be used to identify *de novo* cell-specific enhancers [21]. However, in the absence of sizable *in vivo* validation data sets, the quantitative correlation between tissue-specific eRNA expression and *in vivo* enhancer activity in mammalian developmental processes has remained unclear [21]. We have tested a set of 19 candidate enhancers predicted by tissue-specific RNA expression in transgenic mouse assays and 42% showed reproducible enhancer activity *in vivo*, demonstrating the general utility of eRNA-based enhancer prediction in a developmental mammalian system. Of note, two of the tissue-specific enhancers reported in this study (mm1052 and mm1061) did not overlap with any CAGE peaks collected from 399 mouse samples [30] despite the scope of the tissue and cell type panels examined in these previous studies. Considering the dynamics of the enhancer landscape in developing tissues and organs [29], it appears likely that many additional enhancers active during development will be identifiable by whole transcriptome analysis of tissues across different developmental stages.

While a substantial proportion of extragenic transcription appears linked to enhancer activity, our observation of several TSTRs that were not active in the transgenic enhancer reporter assays supports the hypothesis that eRNA-like transcripts can also originate from other non-coding elements, such as inactive enhancers. These observations are consistent with recent mechanistic studies on eRNAs showing that eRNA transcription precedes the establishment of H3K4me1/2 [38], suggesting that eRNA transcription may occur before enhancer activation. TSTRs without supportive p300/H3K27ac marks show significant, though slightly decreased conservation, less bi-directional transcription, and are more distal to the nearest coding genes (**Figure S3**), suggesting that they may have different biological functions. Consistent with this observation, a larger proportion of TSTRs with supportive p300/H3K27ac marks were active *in vivo* compared to TSTRs without such marks, although this difference was not significant at the sample size examined (p = 0.15, Fisher's exact test; **Table S8**). While the results of our study do not permit strong conclusions about the functionality of intergenic loci that exhibit transcription but no accompanying enhancer epigenomic signatures, it is possible that these regions are less likely to be active enhancers. Transcription may be occurring due to other processes or at a different class of regulatory element than active enhancers. Together, our data suggest that additional criteria such as bi-directional transcription, conservation and independent enhancer marks may further increase the performance of eRNA-based enhancer predictions. Nonetheless, considering the overall substantial correlation between TSTRs and tissue-specific *in vivo* enhancer activity, our results corroborate that short non-coding transcripts are commonly associated with the regulation of cell type- and tissue-specific gene expression.

Enhancer RNAs may be very unstable and sensitive to exosome degradation [21], [39], resulting in low steady-state level in cells. This may explain why eRNAs represent a small proportion of the transcriptome profile (**Figure S2A**), despite the large number of sites from which they originate. At current sequencing depth, many enhancers may still be missed (**Figure S2B**), which is consistent with the notion that a great proportion of mammalian genomes may be actively transcribed and *cis*-regulatory genomic elements may represent major sites of extragenic non-coding transcription [34]–[37], [39]. Recently, Andersson et al. showed that depletion of a co-factor of the exosome complex resulted in an over 3-fold average increase of eRNA abundance [21]. Thus, a combination of in-depth transcriptome profiling and exosome depletion may provide a more sensitive method for eRNA-based enhancer discovery.

Emerging evidence indicates that eRNA transcripts can be required for enhancer-mediated gene activation. Targeted knock-down of specific eRNAs has been shown to affect the expression of enhancer target genes in cell-based assays, providing a potential strategy for altering gene expression in experimental and therapeutic applications [22], [23], [40]. Through in-depth transcriptome profiling, we have shown extensive eRNA expression in developing tissues, as well as a global correlation of eRNA expression with tissue-specific *in vivo* enhancer activity. Our results highlight the widespread and potentially important role of eRNAs in orchestrating gene expression, providing support for the general feasibility of eRNA-based targeting of *in vivo* gene expression.

## Methods

All procedures of this study involving animals were reviewed and approved by the Animal Welfare and Research Committee at Lawrence Berkeley National Laboratory.

### Mouse tissue collection and RNA preparation

Embryonic heart or limb tissue was isolated from CD-1 strain mouse embryos at E11.5 by microdissection in cold PBS [27]. A single sample consisting of tissue pooled from multiple embryos was analyzed for either tissue. After washing, about 1 ml TRIzol reagent (Life Technologies, 15596-026) was added to every 100 mg of tissue sample, followed by homogenization using a glass dounce homogenizer. Total RNA from individual tissues were extracted following the manufacturer's instructions. Genomic DNA contamination was removed by using the TURBO DNA-free kit (Applied Biosystems, AM1907) following manufacture's protocol, and the RNA samples were stored at −80°C before further processing.

### Illumina sequencing of total RNA

In order to perform the transcriptome analysis by Illumina sequencing, ribosomal RNAs was removed from total RNA (5∼10 µg per reaction) by using two rounds of the RiboMinus Eukaryote Kit for RNA-Seq (Life Technologies, A10837-08) following the manufacturer's instructions. The quality of total RNA after rRNA removal was analyzed on RNA 6000 Pico chip (Agilent, 5067-1513) to assure that rRNA contamination was less than 30%. 100 ng total RNA after rRNA removal were used to construct the individual sequencing libraries for Illumina sequencing. Strand-specific RNA-Seq libraries were created following in-house protocols. Briefly, RNA samples were fragmented with 10×Fragment buffer (Ambion, AM9938) to achieve an average fragment size of 200–300 nt. First strand cDNA synthesis was performed with random hexamer and Superscript II reverse transcriptase (Life Technologies, 18064-014). During the second strand synthesis, dUTP was used instead of dTTP to introduce strand-specificity. After adaptor ligation and size selection, the second strand containing dUTP was cleaved by AmpErase UNG (Life Technologies, N8080096). The resulting strand-specific cDNA was subjected to 12 cycles of PCR amplification and sequenced with HiSeq 2000 instrument. 50 sequencing cycles were carried out.

### Data processing and de novo peak calling

Raw Illumina reads (50 bp) were first filtered using the Illumina CASAVA-1.8 FASTQ Filter module (http://cancan.cshl.edu/labmembers/gordon/fastq_illumina_filter/). The remaining sequence tags were mapped back to the mouse genome (NCBI build 37, mm9) using bowtie2 [41], and the alignments were extended to 200 bp in the 3′ direction to account for the average length of DNA fragments. Repetitively mapped reads were excluded from the following analysis. For *de novo* peak calling, a sliding window method EnrichedRegionMaker module from USEQ [42] was employed. For eRNA-based enhancer predictions, a conservative threshold of 10 or more reads (without considering strand specificity) was chosen based on the observation that in retrospective comparison with *in vivo* validated enhancers, 40.7% of enhancers met or exceeded this expression threshold, compared to 4.5% of random control regions (p = 5.5E-108, **Table S2**). Enriched regions overlapping with refGene, mouse mRNA, or ESTs (mm9) were also removed before the downstream analysis. This process was performed individually for heart and limb RNA-Seq data. To generate **Figure S2B**, 10% to 100% of sequencing reads were randomly selected from the raw sequencing data, and *de novo* peak calling was individually performed to identify the enriched intergenic regions.

Among raw enriched regions, tissue-specifically transcribed regions (TSTRs) were defined as non-coding regions with significantly higher expression in this tissue compared with the other tissue (p<0.01, two-proportion z-test; **Figure 3A–B**) [43] with the equation shown below:
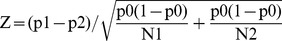
where 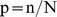 (n represents mappable reads within each TSTR in heart or limb, and N represent the total number of mappable reads excluding ribosomal regions in the corresponding tissue) and 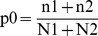. RPKM<2^−9^ were arbitrarily set to 2^−9^ for visualization purposes in **Figure 3A–B**.

Candidate transcription start sites (TSSs) marked by CAGE peaks were downloaded from http://fantom.gsc.riken.jp/5/ [30] and extended to 1 kb each side from the peak midpoint. For each TSTR (1 kb around the peak center), the overlapping candidate TSSs were identified by BEDTools [44]. Random control peaks were also generated using BEDTools with the same number and size of sequences and excluding known genes, mouse mRNAs and ESTs.

### Enhancer predictions based on epigenomic marks

We compared tissue-derived RNA signatures at intergenic regions to enhancer-associated p300 [27] and H3K27ac marks from the same tissues and time-point. H3K27ac ChIP-Seq datasets are described in more detail in Nord et al. [29] and Attanasio et al. [45]. Candidate tissue-specific intergenic enhancers were predicted by ChIP-Seq of p300 (171 in heart, 656 in limb) or H3K27ac (6965 in heart, 2174 in limb) as described previously [27]. Briefly, uniquely aligned sequencing reads were extended to 300 bp in the 3′ direction. Enriched regions (peaks) were identified with MACS [46] (p≤1E-5) using matched input as controls. Peaks overlapping with repetitive regions, known genes, mouse mRNAs and ESTs were removed for further analysis.

### Enhancer RNA coverage

Summary eRNA coverage plots were generated for p300- and/or H3K27ac-derived intergenic enhancers within a 10 kb window, centering on the maximum ChIP-seq coverage. Using the mapped reads, normalized mean eRNA coverage values were calculated for 25 bp windows across the 10 kb regions scaled by total mapped reads. For mean calculations, only the 5^th^–95^th^ percentiles were used to reduce the effect of outliers. Coverage was calculated separately for antisense and sense reads, and as a combined value. For the summary plots, a loess best fit line was plotted for each of the eRNA datasets (limb and heart), separating into sense and antisense reads (**Figure 2C–F**).

### Conservation

Pre-computed conservation scores (phastCons scores) generated from 30 vertebrate genome alignments were download from the UCSC Genome Browser [47]. For each TSTR (1 kb around the peak center), the conservation score was defined as the most highly constrained overlapping phastCons element in the mouse mm9 genome. Random control peaks were generated using BEDTools with the same number and size of sequences and excluding known genes, mouse mRNAs and ESTs [44]. The percentages of TSTRs and random control regions overlapping phastCons elements were plotted in **Figure 4A**.

### Heatmap generation

Tissue-specific TSTRs were classified as enriched in p300 and/or H3K27ac if the relative ChIP-seq coverage was equal to or greater than the 95^th^ percentile of experiment background coverage estimated across 1 Mb of unique sequence. After classification, coverage heatmaps were generated for ChIP-seq data using normalized coverage values, with input corrections. Coverage was plotted for 25 bp windows centered on the peak RNA coverage and extending 25 kb on either side. For plotting purposes, coverage was centered and scaled using mean and SD in order to compare signal across datasets. TSTRs were organized as no H3K27ac and p300 signal, enriched in H3K27ac signal only, enriched in p300 signal only and enriched in both marks from the top to the bottom in **Figure 4C–D**.

### Expression analysis

Known heart or limb enhancers were downloaded from Vista Enhancer Browser (http://enhancer.lbl.gov). For known enhancer regions, the expression level of individual eRNAs was defined as the mapped sequencing reads within a 2 kb window around the center of *in vivo* tested enhancers. For eRNAs only expressed in one tissue, the mapped number of reads was arbitrarily set to 1 in the other tissue in order to compute the absolute fold change for plotting purposes in **Figure 2B**. Fold change was defined as higher expression level divided by lower expression of each eRNA in two tissues. For the volcano plot, y axis represents p-value for the expression differences of each known enhancer, which was computed by two-proportion z-test [43].

Coverage of randomly selected control regions (excluding known genes, mRNA and ESTs) was also computed and iterated 100 times to estimate the genome-wide background based on normal distribution. The percentages of enhancers or the average percentage of control regions with indicated numbers of uniquely mapped reads in either tissue are listed in **Table S2**, as well as associated p-values.

After peak calling, for each individual TSTR, normalized RPKM (Reads Per Kilobase per Million mapped reads) was calculated in two tissues (heart and limb) with the raw mapped RNA-Seq data within a 2 kb window around the center of each TSTR. Then, a tissue-specificity index was computed as (s−u)/(s+u), in which s is the expression of TSTR in the matching tissue and u is its expression in the other tissue. The expression of mouse refGene (mm9) was also analyzed in the same way by computing the RPKM across annotated cDNA regions in two tissues.

The tissue-specific expression correlation between TSTRs and their nearby genes was computed as described [18] with minor modifications. Briefly, we paired each TSTR with the nearby genes. For each set of genes with the same ranked distance to TSTRs (the first to the fifth closest genes), genes were ranked based on tissue-specificity indices and grouped into 20 genes per bin. Average tissue-specificity indices from each bin were used to compute the correlation. The Pearson correlation between nearby genes and the corresponding TSTRs was conducted with the statistics module in the R package (http://cran.r-project.org/).

### Gene ontology analysis

Gene ontology analysis for the genes near TSTR regions was performed by GREAT version 2.02 [48]. Enriched GO biological processes with a binomial p-value and fold enrichment were listed in **Table 1**.

### Motif analysis

For TSTRs in heart and limb, enriched motifs were computed within a 2 kb window around the center of individual TSTRs by the motif finding module of HOMER (Hypergeometric Optimization of Motif EnRichment) [49]. Known motifs for transcription factors with a p-value less than 10^−2^ compared with random genomic sequences were reporter in **Table S5** and **Table S6**.

### Directionality analysis

For directionality analysis, the expression of individual TSTRs in sense and antisense strands was defined as the strand-specific mapped sequencing reads within a 2 kb window around the center of TSTRs in either heart or limb. Then the directionality index was defined as |f−r|/(f+r), in which f is the expression of TSTR in one strand and r is its expression in the other strand in the same tissue.

### Expression validation *in vivo*

Total RNA was extracted from independently collected pools of heart or limb tissues with the same method as described before and synthesized into cDNA by reverse transcription using the SuperScript First-Strand Synthesis System (Invitrogen). Candidate TSTRs for RT-PCR validations were randomly selected from the top 30% differentially expressed regions ranked by Z scores. Expression analysis of candidate TSTRs was carried out by real-time PCR using gene-specific primers (**Table S4**) and KAPA SYBR FAST qPCR Master Mix (KAPA Biosystems) on a Roche LightCycler 480. All primers were designed *in silico* using Primer3 (http://primer3.wi.mit.edu/) and tested for amplification efficiency. Target gene expression was calculated with the 2^−ΔΔC_T_^ method [50] and normalized to the *Gapdh* housekeeping gene.

### *In vivo* transgenic validation

Candidate enhancers for *in vivo* testing were selected randomly from TSTRs with a p-value less than 0.01. The tested regions included up to 2 kb genomic DNA flanking the TSTRs on either sides. This general transgenic procedure has been described before [25], [27]. Briefly, the selected regions were PCR amplified from mouse genomic DNA and cloned into the Hsp68-promoter-LacZ reporter [51], [52]. Genomic coordinates and the PCR primers for the cloned regions are listed in **Table 8**. The transgenic embryos were assayed at E11.5 for expression patterns. A positive enhancer is defined as an element with reproducible expression pattern in at least three embryos resulting from independent transgenic integration events [27]. For histological analysis, selected embryos were embedded in paraffin and sectioned using standard methods.

### Data access

RNA-seq data is available through GEO under accession number GSE58157. *In vivo* transgenic data is available through the Vista Enhancer Browser under the identifiers used throughout this study (http://enhancer.lbl.gov).

## Supporting Information

Figure S1 Example of intragenic eRNA expression from a known intronic enhancer hs1430. Sequencing reads were mapped in a strand-specific manner and displayed separately. Scales corresponding to read count are shown on the left. Genomic region cloned for the transgenic assay is indicated by the green bar. Representative LacZ-stained embryos at E11.5 from transgenic assays for element hs1430 are shown at the bottom. Blue arrowheads indicate reproducible LacZ staining pattern in limb.
(PDF)

Figure S2 Tissue-specific transcription from the extragenic genome. (A) Reads obtained from each tissue-specific total RNA-Seq experiment that unambiguously aligned to the reference mouse genome. Enriched regions/peaks were filtered with annotated gene (including introns, exons and UTRs), mouse mRNA and mouse EST database. (B) Enriched intergenic regions/peaks identified from 10% to 100% of sequencing reads that were randomly selected from raw sequencing data (see **Methods**). (C) Distance distribution between TSTRs in two tissues and their nearest genes. (D) The phastCons conservation scores of heart or limb TSTRs. The scores of the most highly constrained phastCons elements in the mouse genome overlapped with 1 kb regions flanking the center of individual TSTRs were plotted (see **Methods**). For box plot in B and C, upper hinge of the box, lower hinge of the box and horizontal line within the box indicates 75^th^ percentile, 25^th^ percentile and median, respectively. The whiskers represent the minimum and maximum values.
(PDF)

Figure S3 Characteristics of TSTRs with or without enhancer mark(s). (A) Expression of individual TSTRs in heart and limb is shown in box plot. For each TSTR, normalized expression (mapped read count per kb, log10 transformed) was calculated in two tissues (heart and limb) with the raw mapped RNA-Seq data. (B) The fraction of TSTRs (with or without enhancer marks) or random control regions that were under strong evolutionary constraint. One kb flanking the center of TSTRs or control regions were assigned the score of the most highly constrained overlapping 30 vertebrate phastCons scores (see **Methods**). Error bars represent 95% binomial proportion confidence interval. (C and D) Cumulative plot of the directionality index (see **Methods**) in heart (C) and limb (D), respectively. (E) Distance distribution between TSTRs in two tissues and their nearest genes. For box plot in A and E, upper hinge of the box, lower hinge of the box and horizontal line within the box indicates 75^th^ percentile, 25^th^ percentile and median, respectively. The whiskers represent the minimum and maximum values.
(PDF)

Figure S4 Additional TSTRs tested in transgenic assays. For each tested element, lateral views of whole-mount LacZ-stained embryos at E11.5 are shown in top left panels and close-ups of LacZ-positive tissue (black dashed line) are shown in the top right panels. Arrowheads indicate reproducible LacZ staining pattern in limb (blue). The shape of the limb is outlined by a dashed orange line. Element ID and reproducibility of expression patterns are indicated at the bottom of the images. Strand-specific eRNA coverage of tested regions in heart (red) or limb (blue) is show in the bottom panels. Scales corresponding to read count are shown on the left of the coverage. Genomic regions cloned for the transgenic assays are indicated by green bars. (A) Enhancer element mm734. (B) Enhancer element mm757. (C) Enhancer element mm1061. (D) Enhancer element mm1063. Transgenic results of all tested elements are available through the Vista Enhancer Browser (http://enhancer.lbl.gov).
(PDF)

Table S1 Summary of mappability from total RNA-Seq results.
(DOCX)

Table S2 Summary of read counts uniquely mapped to *in vivo* validated enhancers or control regions (100 times iteration).
(DOCX)

Table S3 List of all tissue-specific TSTRs.
(XLSX)

Table S4 List of quantitative RT-PCR primers for the validation of tissue-specific eRNA expression.
(DOCX)

Table S5 Enriched motifs of known transcription factors among heart-specific TSTRs.
(XLSX)

Table S6 Enriched motifs of known transcription factors among limb-specific TSTRs.
(XLSX)

Table S7 Tested elements from Vista Enhancer Browser overlapping TSTRs (mm9).
(DOCX)

Table S8 List of cloning primers for *in vivo* transgenic assays.
(DOCX)
