# Supplementary figures and images for: Tissue-Specific RNA Expression Marks Distant-Acting Developmental Enhancers
Source: PLoS Genet. 2014 Sep 4;10(9):e1004610. doi: 10.1371/journal.pgen.1004610 (PMC4154669; doi:10.1371/journal.pgen.1004610)

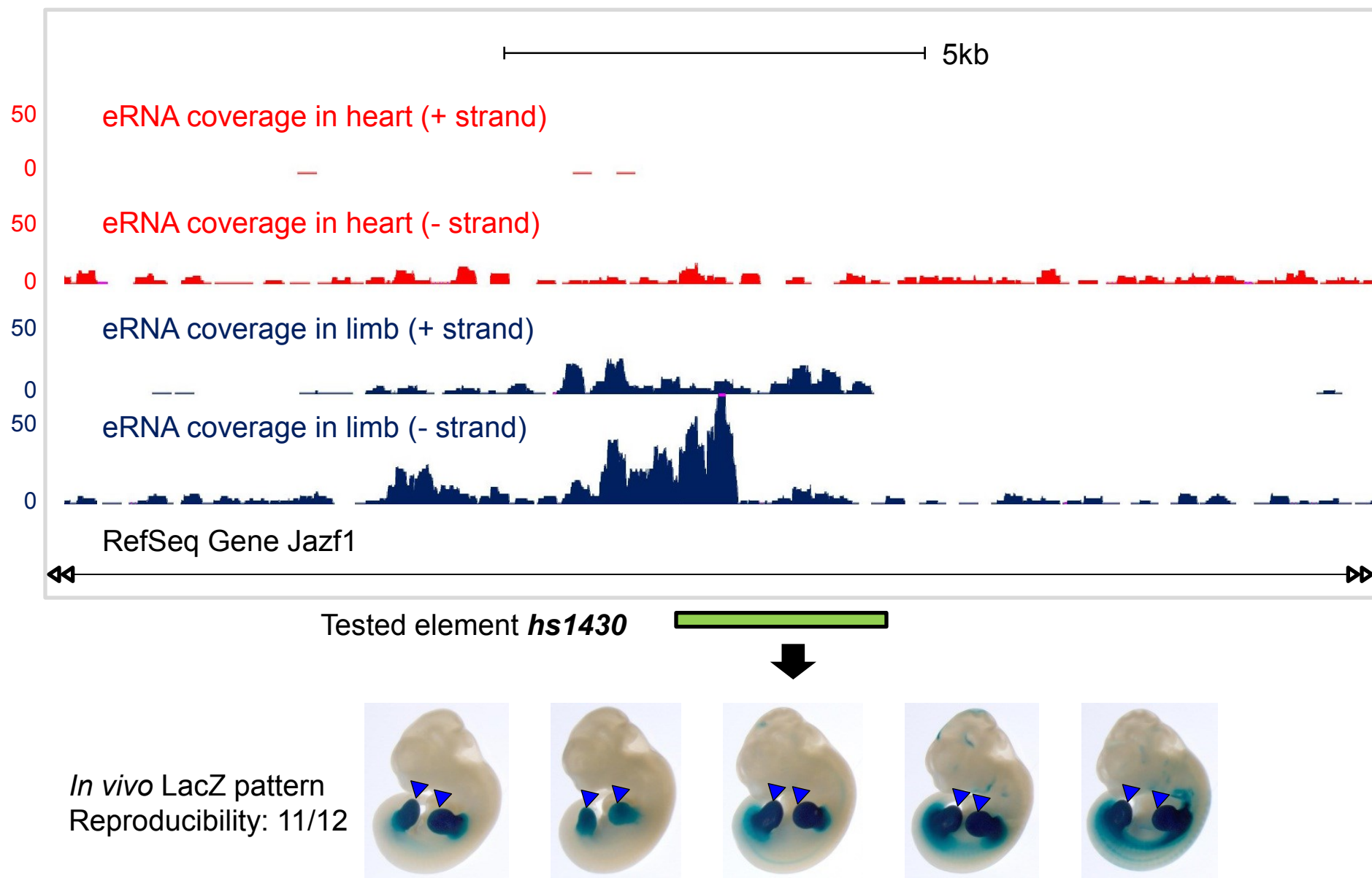

Figure S1

Supplement: Figure S1 — Example of intragenic eRNA expression from a known intronic enhancer hs1430. Sequencing reads were mapped in a strand-specific manner and displayed separately. Scales corresponding to read count are shown on the left. Genomic region cloned for the transgenic assay is indicated by the green bar. Representative LacZ-stained embryos at E11.5 from transgenic assays for element hs1430 are shown at the bottom. Blue arrowheads indicate reproducible LacZ staining pattern in limb. (PDF) [file pgen.1004610.s001.pdf]

A

## Heart peak calling summary

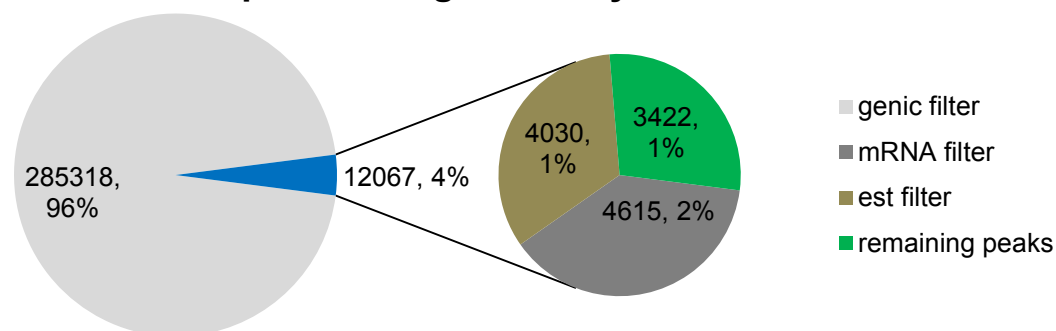

## Limb peak calling summary

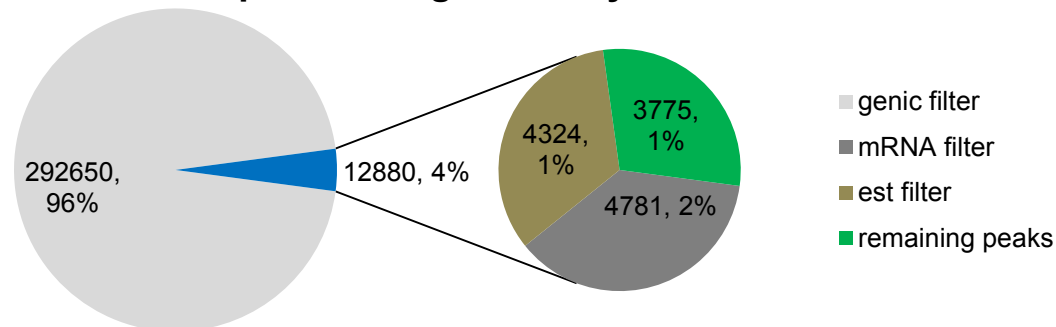

B

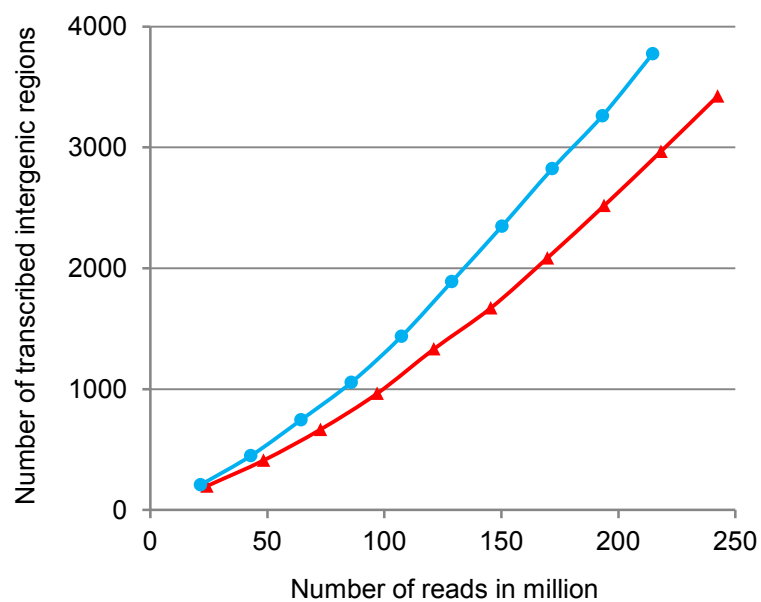

C

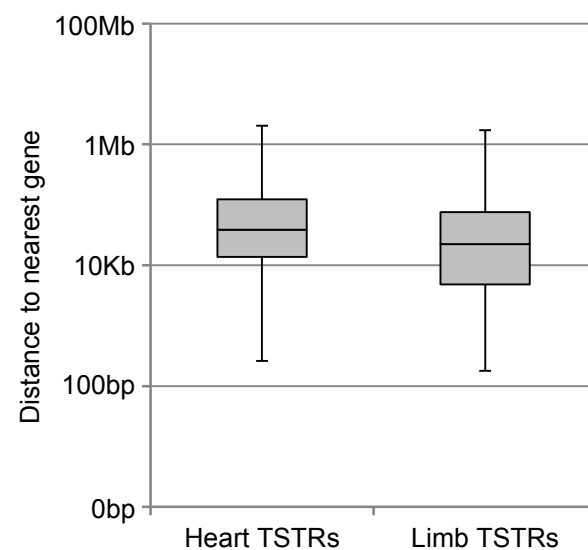

D

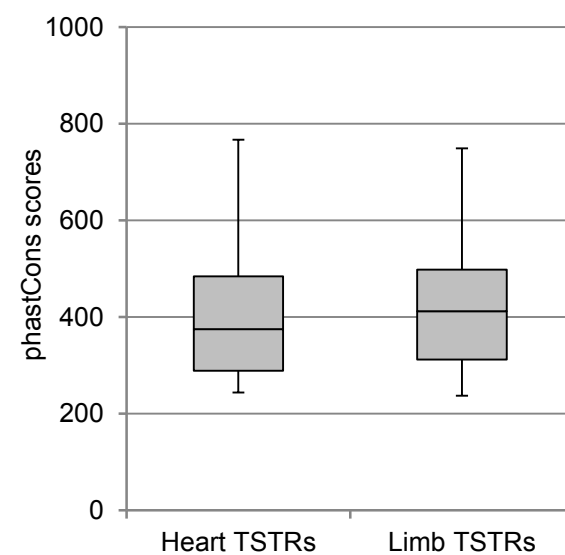

Figure S2

Supplement: Figure S2 — Tissue-specific transcription from the extragenic genome. (A) Reads obtained from each tissue-specific total RNA-Seq experiment that unambiguously aligned to the reference mouse genome. Enriched regions/peaks were filtered with annotated gene (including introns, exons and UTRs), mouse mRNA and mouse EST database. (B) Enriched intergenic regions/peaks identified from 10% to 100% of sequencing reads that were randomly selected from raw sequencing data (see Methods ). (C) Distance distribution between TSTRs in two tissues and their nearest genes. (D) The phastCons conservation scores of heart or limb TSTRs. The scores of the most highly constrained phastCons elements in the mouse genome overlapped with 1 kb regions flanking the center of individual TSTRs were plotted (see Methods ). For box plot in B and C, upper hinge of the box, lower hinge of the box and horizontal line within the box indicates 75th percentile, 25th percentile and median, respectively. The whiskers represent the minimum and maximum values. (PDF) [file pgen.1004610.s002.pdf]

A

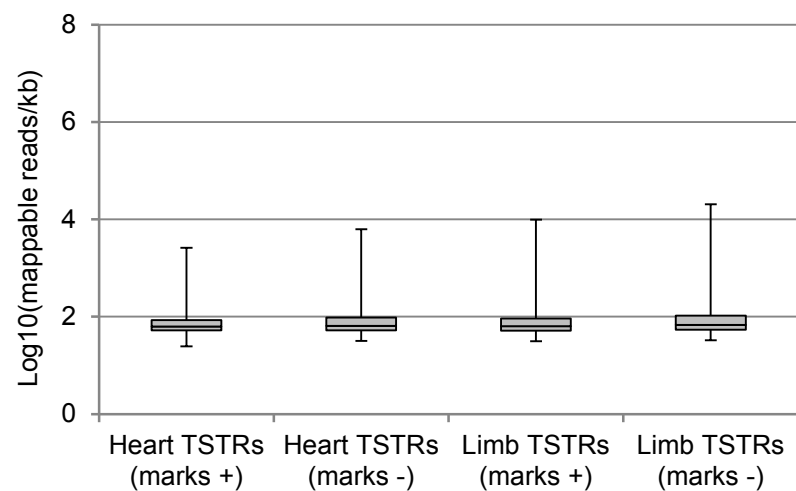

### Center of TSTRs

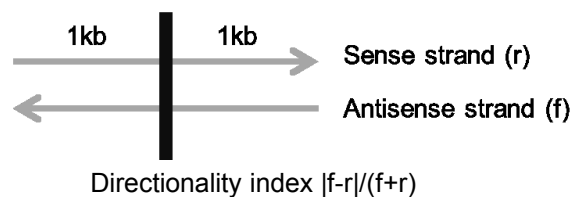

C

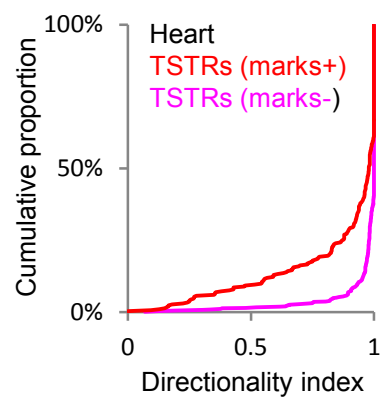

D

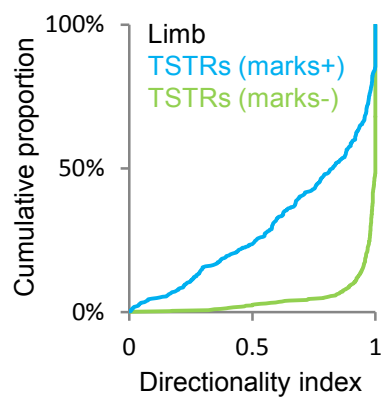

B

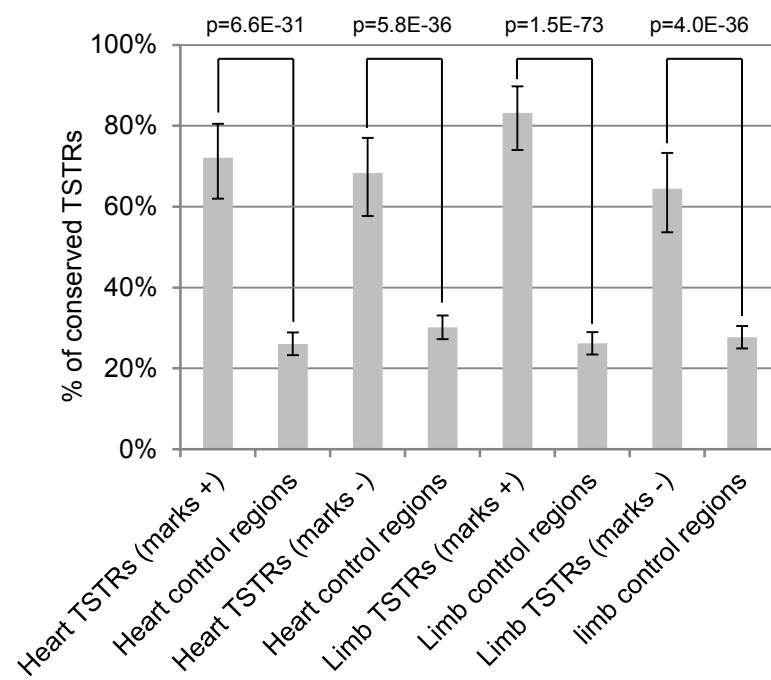

E

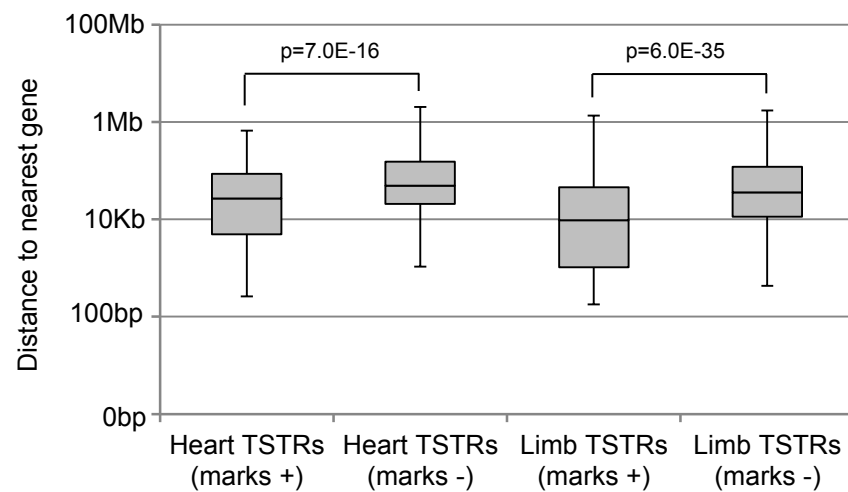

Figure S3

Supplement: Figure S3 — Characteristics of TSTRs with or without enhancer mark(s). (A) Expression of individual TSTRs in heart and limb is shown in box plot. For each TSTR, normalized expression (mapped read count per kb, log10 transformed) was calculated in two tissues (heart and limb) with the raw mapped RNA-Seq data. (B) The fraction of TSTRs (with or without enhancer marks) or random control regions that were under strong evolutionary constraint. One kb flanking the center of TSTRs or control regions were assigned the score of the most highly constrained overlapping 30 vertebrate phastCons scores (see Methods ). Error bars represent 95% binomial proportion confidence interval. (C and D) Cumulative plot of the directionality index (see Methods ) in heart (C) and limb (D), respectively. (E) Distance distribution between TSTRs in two tissues and their nearest genes. For box plot in A and E, upper hinge of the box, lower hinge of the box and horizontal line within the box indicates 75th percentile, 25th percentile and median, respectively. The whiskers represent the minimum and maximum values. (PDF) [file pgen.1004610.s003.pdf]
