# Supplementary material for: Tissue-Specific RNA Expression Marks Distant-Acting Developmental Enhancers
Source: PLoS Genet. 2014 Sep 4;10(9):e1004610. doi: 10.1371/journal.pgen.1004610 (PMC4154669; doi:10.1371/journal.pgen.1004610)
Supplement: Figure S4 — Additional TSTRs tested in transgenic assays. For each tested element, lateral views of whole-mount LacZ-stained embryos at E11.5 are shown in top left panels and close-ups of LacZ-positive tissue (black dashed line) are shown in the top right panels. Arrowheads indicate reproducible LacZ staining pattern in limb (blue). The shape of the limb is outlined by a dashed orange line. Element ID and reproducibility of expression patterns are indicated at the bottom of the images. Strand-specific eRNA coverage of tested regions in heart (red) or limb (blue) is show in the bottom panels. Scales corresponding to read count are shown on the left of the coverage. Genomic regions cloned for the transgenic assays are indicated by green bars. (A) Enhancer element mm734. (B) Enhancer element mm757. (C) Enhancer element mm1061. (D) Enhancer element mm1063. Transgenic results of all tested elements are available through the Vista Enhancer Browser (http://enhancer.lbl.gov). (PDF) [file pgen.1004610.s004.pdf]

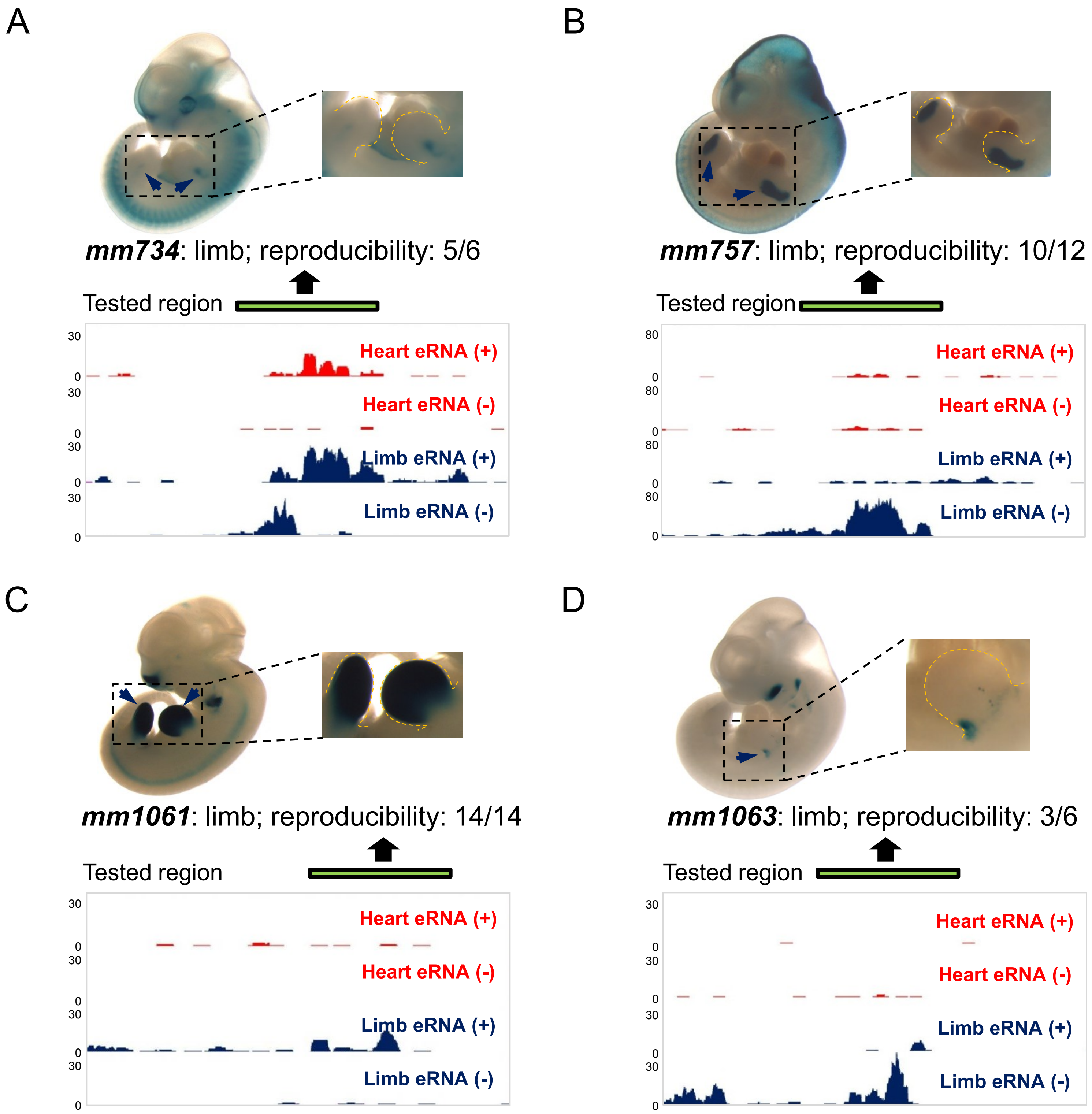
A
mm734: limb; reproducibility: 5/6
Tested region
Heart eRNA (+)
Heart eRNA (-)
Limb eRNA (+)
Limb eRNA (-)
30
0
B
mm757: limb; reproducibility: 10/12
Tested region
Heart eRNA (+)
Heart eRNA (-)
Limb eRNA (+)
Limb eRNA (-)
80
0
C
mm1061: limb; reproducibility: 14/14
Tested region
Heart eRNA (+)
Heart eRNA (-)
Limb eRNA (+)
Limb eRNA (-)
30
0
D
mm1063: limb; reproducibility: 3/6
Tested region
Heart eRNA (+)
Heart eRNA (-)
Limb eRNA (+)
Limb eRNA (-)
30
0

Figure S4
