# Supplementary material for: Tissue-Specific RNA Expression Marks Distant-Acting Developmental Enhancers
Source: PLoS Genet. 2014 Sep 4;10(9):e1004610. doi: 10.1371/journal.pgen.1004610 (PMC4154669; doi:10.1371/journal.pgen.1004610)
Supplement: Table S1 — Summary of mappability from total RNA-Seq results. (DOCX) [file pgen.1004610.s005.docx]

**Table S1: Summary of mappability from total RNA-Seq results**

| **Tissue** | **Heart** | **Limb** |
| --- | --- | --- |
| ***total # of reads*** | 242,542,843 | 214,671,051 |
| ***rRNA %*** | 26.9 | 30.7 |
| ***unique mappable %*** | 53.3 | 50.5 |
| ***genome mappable %*** | 91.7 | 89.7 |
| ***mapped to cDNA %*** | 53.4 | 60.0 |
| ***mapped to other regions %*** | 38.2 | 29.7 |
