# Supplementary material for: Tissue-Specific RNA Expression Marks Distant-Acting Developmental Enhancers
Source: PLoS Genet. 2014 Sep 4;10(9):e1004610. doi: 10.1371/journal.pgen.1004610 (PMC4154669; doi:10.1371/journal.pgen.1004610)
Supplement: Table S2 — Summary of read counts uniquely mapped to in vivo validated enhancers or control regions (100 times iteration). (DOCX) [file pgen.1004610.s006.docx]

**Table S2: Summary of read counts uniquely mapped to *in vivo* validated enhancers or control regions (100 times iteration)**

| **# of uniquely mapped sequencing reads** | **≥ 1** | **≥ 2** | **≥ 5** | **≥ 10** |
| --- | --- | --- | --- | --- |
| ***% of positive enhancers*** | 91.7% | 77.2% | 56.6% | 40.7% |
| ***average % of random control regions*** | 60.1% | 35.4% | 11.5% | 4.5% |
| ***p-value*** | 2.3E-15 | 3.2E-26 | 7.7E-73 | 5.5E-108 |
