# Supplementary material for: Tissue-Specific RNA Expression Marks Distant-Acting Developmental Enhancers
Source: PLoS Genet. 2014 Sep 4;10(9):e1004610. doi: 10.1371/journal.pgen.1004610 (PMC4154669; doi:10.1371/journal.pgen.1004610)
Supplement: Table S4 — List of quantitative RT-PCR primers for the validation of tissue-specific eRNA expression. (DOCX) [file pgen.1004610.s008.docx]

**Table S4: List of quantitative RT-PCR primers for the validation of tissue-specific eRNA expression**

| **Element** | **Name** | **Sequence** | **Amplicon (bp)** |
| --- | --- | --- | --- |
| ***limb enhancer hs1473*** | **hs1473_F** | CACCCTTCTCTGGCTAAGGA | 102 |
|  | **hs1473_R** | CCAGGACAACCATGATTCAA |  |
| ***limb enhancer hs1434*** | **hs1434_F** | CTGCTCAGACACAGGTTTCC | 135 |
|  | **hs1434_R** | GTGTTTGTTAGTTCTCTGCAAGG |  |
| ***limb enhancer mm429*** | **mm429_F** | TCACCCAACCTTTCATTTCTC | 86 |
|  | **mm429_R** | AAAGGGACTCACAGCCCTAA |  |
| ***limb enhancer hs1431*** | **hs1431F** | CCTCACAGAAGCATTTGTCC | 103 |
|  | **hs1431R** | TGCTGTCACGGATACCTCAT |  |
| ***limb enhancer mm466*** | **mm466F** | AGCAGGGCCAATCTGTTTAC | 144 |
|  | **mm466R** | CACATCTGGATGGAGTCAGC |  |
| ***limb enhancer hs1620*** | **hs1620F** | AGGACTTTCAGCCCTCAGAA | 105 |
|  | **hs1620R** | ATGCCTGAAGTGCATGTGTT |  |
| ***limb enhancer mm734*** | **mm734F** | GAGGAAATCAAACCAGCGTT | 130 |
|  | **mm734R** | CCCTCCTTCTGACTCTCAGG |  |
| ***limb enhancer hs1437*** | **hs1437_F** | GGACAGGTGAGGGCATTT | 96 |
|  | **hs1437_R** | CATTTACCTCTGCCCATCTG |  |
| ***forebrain enhancer mm505*** | **mm505_F** | GTCAAGAATGCTTGGAATGC | 114 |
|  | **mm505_R** | CCCTCTATCCAGTCCCAGAG |  |
| ***forebrain enhancer hs1743*** | **hs1743_F** | CTTTGTGCAAGCTTTGCTGT | 110 |
|  | **hs1743_R** | GGCATCAAGACATCCAGAGA |  |
| ***forebrain enhancer hs1325*** | **hs1325_F** | GGACTGGCAGGGAAAGACTA | 120 |
|  | **hs1325_R** | ACAGCAACCCAAACAAGGTT |  |
| ***forebrain enhancer mm745*** | **mm745_F** | AGGAGAATAAAGCCCTTCCC | 108 |
|  | **mm745_R** | TGGCTAGACAGACACCAAGC |  |
| ***forebrain enhancer mm13*** | **mm13_F** | CGCAAACACTATGTCATTCCA | 131 |
|  | **mm13_R** | ACAAGGCCGTTGCACTTAC |  |
| ***forebrain enhancer mm569*** | **mm569_F** | GAGATTTAGCGCATGACGAA | 129 |
|  | **mm569_R** | ATCCTACTCCGGGTCCTCTT |  |
| ***forebrain enhancer hs1302*** | **hs1302_F** | TGATATGAGGTGGTGGGAAA | 108 |
|  | **hs1302_R** | CCATCAATTGCTGTGGTCAT |  |
| ***heart TSTR 33*** | **heart_33_F** | GGGCAGTGATCCTGCTATTT | 107 |
|  | **heart_33_R** | GTTCCACAAAGCAAGCAGAA |  |
| ***heart TSTR 361*** | **heart_361_F** | ACTTCCTCTCCCGGGTATTT | 148 |
|  | **heart_361_R** | GCTGGCTGTCAGAGATTCAA |  |
| ***heart TSTR 440*** | **heart_440_F** | CGACATCATGCTGAAGGAAT | 133 |
|  | **heart_440_R** | CTTTACAAGTGGTGACAGCAAA |  |
| ***heart TSTR 659*** | **heart_659_F** | TGGCTTCTGACATCTCCAAC | 142 |
|  | **heart_659_R** | TGAAGATGACCATGGAAGGA |  |
| ***heart TSTR 1123*** | **heart_1123_F** | TTGCAATGGCAACTGAAAGT | 118 |
|  | **heart_1123_R** | CCCATTGTTCACATGAGGAG |  |
| ***heart TSTR 1138*** | **heart_1138_F** | GGGAACATGTGGCTGTAGTG | 140 |
|  | **heart_1138_R** | CACATACGGGTGACTCCAAG |  |
| ***heart TSTR 1351*** | **heart_1351_F** | CCTGGTTGAAGTCCATTCCT | 113 |
|  | **heart_1351_R** | TGCTCCCTGTTTAATCCCTT |  |
| ***heart TSTR 1354*** | **heart_1354_F** | TTATCATGGCAGCAGAAAGG | 148 |
|  | **heart_1354_R** | TGACAACAAGAGGGCTTGAG |  |
| ***heart TSTR 1648*** | **heart_1648_F** | GCTATCACTTTGCCCACAGA | 135 |
|  | **heart_1648_R** | CAGGAAACAAGACCAAGCAA |  |
| ***heart TSTR 1676*** | **heart_1676_F** | TTTCGTGAATTCTTCAAACCA | 149 |
|  | **heart_1676_R** | CAGAGACAACCAAGTTGCTGA |  |
| ***heart TSTR 2189*** | **heart_2189_F** | GGGTGCTCAGGGTGTTAGTT | 136 |
|  | **heart_2189_R** | TCCTAAACCAGGTGAATCCC |  |
| ***heart TSTR 2241*** | **heart_2241_F** | GGCGCTAACAAAGACTTCCT | 150 |
|  | **heart_2241_R** | TTGTCAGCAGTGTTTCAGCA |  |
| ***limb TSTR 208*** | **limb_208_F** | AGAGGCAAGGCATCTCTCAT | 149 |
|  | **limb_208_R** | TTAGCAGATTTGCTGATGGG |  |
| ***limb TSTR 361*** | **limb_361_F** | ACTTCAAGTCTGCTGGCCTT | 118 |
|  | **limb_361_R** | TCCCTGTGATCAATGCAAAT |  |
| ***limb TSTR 365*** | **limb_365_F** | GATGTCCCAGTGCGTAAATG | 147 |
|  | **limb_365_R** | GCCTGGTATGAGATGTGTGC |  |
| ***limb TSTR 366*** | **limb_366_F** | GATCACCCTTCAAAGGCACT | 109 |
|  | **limb_366_R** | TGCCATCCAGAACTCATCAT |  |
| ***limb TSTR 678*** | **limb_678_F** | ACAAGAGAAGGCAGGAGGAA | 146 |
|  | **limb_678_R** | CAGGGTGGCTTTCTGTCTCT |  |
| ***limb TSTR 1001*** | **limb_1001_F** | CCTGCCCATATTGCTTACTTC | 112 |
|  | **limb_1001_R** | GACGCGTGTTCTTCTCAGC |  |
| ***limb TSTR 1180*** | **limb_1180_F** | TTAGACCAGGACACCCAACA | 133 |
|  | **limb_1180_R** | CTCCCACTGACATCATTGCT |  |
| ***limb TSTR 1238*** | **limb_1238_F** | AGCATCAGCCAATCATTCAC | 129 |
|  | **limb_1238_R** | GCTTGTGCAGAAGGAATCAA |  |
| ***limb TSTR 1255*** | **limb_1255_F** | ACGCTTCTGGCAATGTGTAG | 144 |
|  | **limb_1255_R** | GCCTTGAGCACAAGATGAAG |  |
| ***limb TSTR 2460*** | **limb_2460_F** | GCTGGACTCAGAGGACTTGG | 143 |
|  | **limb_2460_R** | TCTAATGCTGACCGTTTCTCA |  |
