# Supplementary material for: Tissue-Specific RNA Expression Marks Distant-Acting Developmental Enhancers
Source: PLoS Genet. 2014 Sep 4;10(9):e1004610. doi: 10.1371/journal.pgen.1004610 (PMC4154669; doi:10.1371/journal.pgen.1004610)
Supplement: Table S7 — Tested elements from Vista Enhancer Browser overlapping TSTRs (mm9). (DOCX) [file pgen.1004610.s011.docx]

**Table S7: Tested elements from Vista Enhancer Browser overlapping TSTRs (mm9)**

| **Element** | **Chr** | **Start** | **End** | **Enhancer Annotation** |
| --- | --- | --- | --- | --- |
| ***mm77*** | chr5 | 122,542,227 | 122,544,777 | heart |
| ***mm771*** | chr14 | 55,615,731 | 55,616,061 | heart |
| ***mm85*** | chr11 | 65,462,408 | 65,465,145 | heart |
| ***mm213*** | chr11 | 68,436,977 | 68,437,676 | heart and others |
| ***mm253*** | chr5 | 38,900,507 | 38,901,138 | heart |
| ***mm425*** | chr5 | 38,209,891 | 38,210,884 | neural tube |
| ***mm406*** | chr6 | 50,933,966 | 50,934,809 | limb and others |
| ***mm426*** | chr5 | 38,216,908 | 38,220,459 | limb and others |
| ***mm260*** | chr12 | 86,795,350 | 86,797,239 | heart |
| ***mm409*** | chr14 | 35,007,307 | 35,009,236 | negative |
| ***mm59*** | chr1 | 167,977,609 | 167,981,010 | negative |
| ***mm370*** | chr9 | 41,697,093 | 41,700,596 | negative |
