# Supplementary material for: Tissue-Specific RNA Expression Marks Distant-Acting Developmental Enhancers
Source: PLoS Genet. 2014 Sep 4;10(9):e1004610. doi: 10.1371/journal.pgen.1004610 (PMC4154669; doi:10.1371/journal.pgen.1004610)
Supplement: Table S8 — List of cloning primers for in vivo transgenic assays. (DOCX) [file pgen.1004610.s012.docx]

**Table S8: List of cloning primers for *in vivo* transgenic assays**

| **Element** | **Primer** | **Sequence** | **Amplicon (bp)** | **H3K27ac or p300 signal** | **In vivo result** |
| --- | --- | --- | --- | --- | --- |
| ***mm734*** | **F** | CACCTGCAGCCATTAAAGGCAT | 3504 | yes | (5/6) limb |
|  | **R** | ACTCCCTCCCAACTTTGACA |  |  |  |
| ***mm757*** | **F** | CACCCATCTCAGCCATACCTTT | 3402 | yes | (10/12) limb |
|  | **R** | TGACTAAGGAGTTTAGAGCACACA |  |  |  |
| ***mm1018*** | **F** | CACCACAGCCAAGGTTGTCCTCTG | 4,106 | yes | (5/7) heart |
|  | **R** | TGCCTTTTGGAACATGAAGA |  |  |  |
| ***mm1051*** | **F** | CACCATGTGCTCCTCTCTGCTGGT | 3,347 | yes | neg |
|  | **R** | TCAGTTTGGATGAAAAGGTGATT |  |  |  |
| ***mm1052*** | **F** | CACCGGCAAGCAGACAGTGTTTCA | 3,374 | yes | (10/10) heart |
|  | **R** | CTACCCCATTGACCCTTTCA |  |  |  |
| ***mm1053*** | **F** | CACCGCTACACCCAGGAAGAGCTG | 3,463 | yes | neg |
|  | **R** | CAATGCTGATGGTGTTACGG |  |  |  |
| ***mm1054*** | **F** | CACCCACAAGCACTGGCTCAAAAA | 4,396 | yes | (10/10) heart |
|  | **R** | TTCATCTCCATGCATTTCCA |  |  |  |
| ***mm1055*** | **F** | CACCAAACACCTGGGGAGTGACAG | 3,613 | yes | neg |
|  | **R** | CTTTTAAACGCCCCAATGAA |  |  |  |
| ***mm1056*** | **F** | CACCACAGGACTTGATTGGGCAAC | 3,427 | no | neg |
|  | **R** | TTCATGCTAGCCCAATACCC |  |  |  |
| ***mm1058*** | **F** | CACCCCAGCAGAACCTGTGTAGCA | 4,322 | yes | neg |
|  | **R** | GACCTTTGTGTGAACGCTGA |  |  |  |
| ***mm1061*** | **F** | CACCAAGCCCTCTTGCTGCAAATA | 3,639 | yes | (14/14) limb |
|  | **R** | GGGATTCGCCTAAACAAACA |  |  |  |
| ***mm1062*** | **F** | CACCTGGCTCAGCAAGGAAAAAGT | 3,472 | yes | neg |
|  | **R** | CAAAGCATTTTGTGGGTGTG |  |  |  |
| ***mm1063*** | **F** | CACCATGTCCAGGCTAAGGGTGTG | 3,821 | yes | (3/6) limb |
|  | **R** | GCTGGGTGGAAAAGTGTTGT |  |  |  |
| ***mm1064*** | **F** | CACCGTGTCCGCTTGCTCTCTACC | 3,507 | no | (3/5) limb |
|  | **R** | TAAAGCCAGGCTGCTTCATT |  |  |  |
| ***mm1071*** | **F** | CACCCAGAGGTGGCCAGTTCAAT | 3,982 | no | neg |
|  | **R** | TGCAGTGCAAGTGTTTATGG |  |  |  |
| ***mm1073*** | **F** | CACCCAGGTGAGAAGGGTGAGAGC | 4,151 | no | neg |
|  | **R** | CCAAGGACCCACAACCTAGA |  |  |  |
| ***mm1076*** | **F** | CACCCCCAGAAACTGCGATGAAAT | 3,063 | no | neg |
|  | **R** | CAGGAACAGAAGGTGGGGTA |  |  |  |
| ***mm1077*** | **F** | CACCCCCAGGGTCTGATGTGAAGT | 3,030 | yes | neg |
|  | **R** | GTAGCTTTGGGTGCTTCCTG |  |  |  |
| ***mm1082*** | **F** | CACCCCTAATCCCCCTCTCCAAAC | 3,001 | no | neg |
|  | **R** | AACAACAATGGAGGCGAAAC |  |  |  |
